# Prognostic value of modified shock index in sepsis

**DOI:** 10.6026/973206300210614

**Published:** 2025-04-30

**Authors:** Sanchit Uppal, Mini Bhatnagar, Abhinav Meelu, Johnly Jacob, Ena Sharma

**Affiliations:** 1 Department of General Medicine, Maharishi Markandeshwar Institute of Medical Sciences and Research, Mullana, Ambala, Haryana-133203, India; 2 Department of Periodontics, Rayat Bahra Dental College and Hospital, Mohali, Punjab-140301, India

**Keywords:** Triage, emergency department, quick sequential organ failure assessment (qSOFA), modified shock index (MSI), sepsis

## Abstract

Sepsis is a life-threatening condition requiring early diagnosis and severity assessment in the emergency department (ED) to guide timely interventions. Therefore, it is of interest to evaluate the Modified Shock Index (MSI) as a prognostic tool for sepsis and compared its sensitivity and specificity to the quick sequential organ failure assessment (qSOFA) score in 150 sepsis patients, based on Sepsis-3 guidelines. Clinical data, including MSI and qSOFA scores, were collected at admission and patient outcomes were analyzed for mortality and hospital stay duration. An MSI cut-off of 1.28 predicted mortality with high accuracy (AUC 0.946, sensitivity 91.76%, specificity 90.77%), outperforming the qSOFA score (AUC 0.840). MSI is a reliable, easy-to-use bedside tool that surpasses the quick sequential organ failure assessment score in predicting sepsis prognosis, aiding in early identification and management of high-risk patients.

## Background:

Sepsis is a severe immune response to infection causing organ damage, high morbidity and mortality, particularly in vulnerable populations and can progress to septic shock with fatality rates exceeding 60% [1]. The Third International Consensus (Sepsis-3) defines sepsis as "organ dysfunction is resulting from dysregulated host responses to infection"; in which organ dysfunction is indicated by rise in Sequential Organ Failure Assessment (SOFA) score by 2 or more points in the setting of confirmed or suspected infection [2]. Various scoring systems like SOFA score, Rapid Emergency Medicine Score (REMS), Mortality Prediction Model (MPM) and Acute Physiology and Chronic Health Evaluation (APACHE), assess sepsis prognosis but rely on extensive lab data, while the Quick Sequential Organ Failure Assessment (qSOFA) score, which evaluates systolic blood pressure, respiratory rate and mental status changes, is widely employed in sepsis patients but has limited sensitivity in Emergency Department triage [3]. The Shock Index (SI) which is obtained by dividing heart rate by systolic blood pressure (HR/SBP) and Modified Shock Index (MSI) obtained by dividing heart rate by mean arterial pressure (HR/MAP) offer bedside assessment using basic clinical measurements of sepsis patients [4]. MSI is a valuable prognostic marker in sepsis, aiding in timely diagnosis, fluid resuscitation and vasopressor titration by reflecting vasodilation, low MAP and reduced organ perfusion [5]. Therefore, it is of interest to assess the sensitivity and specificity of the Modified Shock Index (MSI) in predicting the prognosis of sepsis patients during initial triage in the hospital emergency department and to compare it with Quick Sequential Organ Failure Assessment (qSOFA) score.

## Materials and Methods:

This prospective observational study was conducted at a tertiary care medical college in rural North India over a one-year period, from May 1, 2023, to April 30, 2024. Prior approval was obtained from the Institutional Ethical Committee. A total of 150 patients who were diagnosed with sepsis on the basis of sepsis-3 guidelines were selected from the medical emergency department. Patients under 18 years of age, pregnant women, trauma cases and those with surgical causes of sepsis were excluded. After taking written informed consent, participants underwent a detailed clinical history, physical examination and necessary blood investigations. The qSOFA score, which consists of respiratory rate ≥22/min, change in mental status and systolic blood pressure ≤100 mmHg and the Modified Shock Index (MSI), calculated by dividing the heart rate by the mean arterial pressure (MAP), were determined at the time of admission. Patients were monitored throughout their hospital stay and their prognosis was determined based on the duration of hospital stay and mortality. Statistical Analysis: Data collection was performed using a semi-structured, pre-validated preform and the collected data were digitized in MS-Excel. Statistical calculations were conducted using SPSS Version 20. Descriptive statistics, including range, mean ± standard deviation (SD), frequencies and percentages, were used to describe the data. The Kolmogorov-Smirnov test was applied to test the normal distribution of continuous variables. Categorical data, such as the outcome variable 'condition at discharge,' were compared using the Chi-square (χ^2^) test and Fisher's exact test, as appropriate. Independent sample means were compared using the student's t-test. The mean differences in the length of hospital stay for various MSI ranges were analysed using ANOVA followed by post-hoc multiple comparisons to identify significant differences. An ROC curve was constructed for MSI and qSOFA scores to determine the optimal cut-off values. The sensitivity and specificity of these new cut-off values were then evaluated concerning the condition at discharge. Conclusions and statistical inferences were summarized with textual explanations and supported by pictorial and graphical representations to enhance understanding.

## Results:

The study population comprised 150 individuals diagnosed with sepsis based on the Sepsis-3 guidelines, with patient characteristics detailed in Table 1. Using analysis of variance (ANOVA) to compare the mean MSI among the outcome groups, the sum of squares was 25.34 with 2 degrees of freedom, yielding a mean square of 12.670. The high F-value of 47.165 and p-value of 0.001 indicate significant differences among the groups in terms of outcomes (Figure 1 - see PDF). The ROC curve for the MSI in sepsis patients, with a cut-off of 1.28, illustrates the test's accuracy by plotting sensitivity against 1-specificity. The area under the curve (AUC) is 0.946, indicating excellent predictive ability. The ROC curve for qSOFA, with a cut-off of 2, shows its predictive accuracy for patient outcomes, with an area under the curve (AUC) of 0.840. Among patients with an MSI > 1.28, 89.4% (59 out of 66) died, compared to 7.1% (6 out of 84) of those with an MSI < 1.28. Additionally, 80.3% (53 out of 66) of patients with a qSOFA score > 2 died, whereas only 15.5% (13 out of 84) of patients with a qSOFA score < 2 died. The MSI in sepsis patients demonstrates high sensitivity (91.76%) and specificity (90.77%), while the qSOFA for sepsis screening shows sensitivity of 84.52% and specificity of 80.30% (Table 2).

## Discussion:

Despite the recent advances in emergency care, sepsis remains a global health challenge, causing around 6 million deaths annually and ranking as one of the costliest hospital-treated conditions [6]. Early detection and severity assessment are vital for effective care. In low-income countries, high mortality rates stem from underfunded healthcare systems, limited medications and lack of advanced diagnostics and ICU facilities, especially at the primary care level. Delayed diagnosis and treatment often result in critical time lost during patient transfers. Prompt diagnosis and risk classification improve outcomes and help prioritize resources [7]. Predictive scoring systems play a key role in improving outcomes by aiding rapid decision-making. For instance, the qSOFA and MSI scores used in this study are examples of such systems. The MSI was developed to incorporate MAP rather than depending only on SBP. A major loss of vascular tone is a key pathophysiological feature of septic shock in adults and the combination of gradual diastolic hypotension with tachycardia can suggest more severe vasodilatory conditions. Therefore, MSI is more effective in assessing treatment effects for hemodynamic optimization, prognostication and triaging in the emergency department [8]. The present study involving 150 patients, aimed to validate the Modified Shock Index (MSI) as a prognostic indicator for sepsis. Participants ranged from 19 to 97 years, with the largest group aged 66-75 years (25.3%) and an average age of 57.84 ± 16.52 years. Gender distribution was nearly equal (50.7% male, 49.3% female). Comorbidities were present in 76.7% of patients, with hypertension (47.8%) and diabetes mellitus (47%) being the most common. These results align with studies by Ruangsomboon *et al.* [9] and Hammond *et al.* [10], which similarly found high rates of hypertension and diabetes among sepsis patients. Respiratory infections, primarily pneumonia, were the leading cause of sepsis (43.3%), followed by genitourinary (38.7%) and gastrointestinal infections (10.7%), consistent with study by Swain *et al.* [11]. No growth was seen in 39.4% of cultures, likely due to prior antibiotics. Klebsiella pneumoniae was the most common pathogen (24%), with gram-negative bacteria making up 84.1% of isolates, aligning with Chatterjee *et al.* [12] findings. Among the 150 sepsis patients, 122 (81.3%) required ICU admission, while 28 (18.7%) were admitted to the ward. Additionally, 77 patients needed inotropic support and 73 required mechanical ventilation. Overall, higher MSI scores were significantly associated with ICU admissions, while lower scores correlated with ward admissions, underscoring the importance of MSI in determining the appropriate level of care. Research by Paary *et al.* [13] reported similar findings, with a significant proportion of severe sepsis patients requiring mechanical ventilation and inotropic support. Out of 150 sepsis patients, 66 (44%) died, while 84 (56%) survived, aligning with mortality rates of 44% to 52.8% reported in studies by Rhee *et al.* [14] and Ojeda *et al.* [15]. In this study, patients who died within 48 hours had notably higher MSI values compared to those who survived longer, indicating that elevated MSI is linked to rapid deterioration and early death. This highlights the potential of MSI as a prognostic marker for critically ill patients. Higher mortality rates were associated with factors such as advanced age, comorbidities, illness severity and limited ICU resources. A significant association was found between comorbidities and patient outcomes (p=0.001), with a higher mortality rate among those with comorbidities (53.1%) compared to those without (14.2%). Diabetes and COPD were notably associated with worse outcomes, likely due to impaired immunity. Elevated glucose levels in diabetes increase infection risk, while COPD patients often have poor lung function and a history of exacerbations. These factors heighten susceptibility to sepsis. Similar findings have been reported in study by Prasad *et al.* [16], identifying diabetes as a key risk factor for sepsis-related mortality. Patients who died had significantly lower systolic and diastolic blood pressures, higher respiratory rates, lower oxygen saturation and reduced mean arterial pressure. These parameters are indicative of hypotension, tachypnoea, hypoxemia and impaired neurological function, all associated with higher mortality. Study by Liu *et al.* [17] corroborates these findings, emphasizing the importance of vital signs in predicting sepsis outcomes. The mean MSI was 1.41 ± 0.65, with 37 patients (24.7%) having an MSI < 1.0, 52 patients (34.7%) falling between 1.0 and 1.3 and 61 patients (40.7%) having an MSI > 1.3. Statistically significant correlation was found between high MSI and poor outcomes like mortality and longer hospital stay. An MSI cut-off of 1.28 was determined to be the optimal threshold for predicting mortality, with an area under the curve (AUC) of 0.946, indicating high predictive accuracy. This is supported by studies such as those by Prasad *et al.* [16] and Smischney *et al.* [18], which also found MSI to be a reliable prognostic indicator. According to this study, the Modified Shock Index (MSI) outperforms the qSOFA score in several key areas. MSI shows higher sensitivity (91.76% vs. 84.52%), making it better at correctly identifying patients with the condition and higher specificity (90.77% vs. 80.30%), improving its accuracy in ruling out patients without the condition. Similarly, Althunayyan *et al.* [4] identified an MSI ≥1.3 as strongly linked to sepsis, hyperlactatemia, ICU admission and 28-day mortality, with specificity of 59% to 100%. Our study findings suggest that the MSI is a more effective tool for the early detection and management of sepsis patients, offering higher sensitivity, specificity and accuracy in identifying those at risk of adverse outcomes. To our knowledge, this is the first study to compare the statistical parameters of the Modified Shock Index (MSI) and qSOFA in sepsis patients. This study, conducted at Maharishi Markandeshwar Institute of Medical Sciences and Research, Mullana, offers key insights into the utility of qSOFA and MSI scores. By evaluating their sensitivity and specificity against patient outcomes, these findings can help refine clinical protocols and enhance care. Given its simplicity, MSI could serve as a valuable prognostic tool for identifying patients who need referral from primary healthcare facilities to higher centres, improving timely management and overall outcomes.

## Limitations:

One of the study's shortcomings is that it was carried out at a single site with a limited sample size. While it documented important findings, these could be strengthened through larger population studies and multicentre research.

## Conclusion:

The Modified Shock Index (MSI) is a valuable bedside tool for predicting sepsis prognosis, demonstrating high sensitivity, specificity and predictive value, making it a reliable tool for assessing the severity of illness and guiding clinical decision-making. It outperforms the qSOFA score in assessing sepsis severity and guiding clinical decisions. Implementing MSI can improve early identification and management of high-risk sepsis patients, enhancing outcomes and reducing mortality.

## Figures and Tables

**Table 1:** Patient characteristics

**Total patients: 150 Age (mean ± SD) -57.84 ± 16.52 years**		
**Patient Characteristics**	**Number**	**Percentage**
Male	76	50.7
Female	74	49.3
Hypertension	55	47.8
Diabetes mellitus	54	47
Chronic kidney disease	24	20.9
Coronary artery disease	14	12.2
Chronic obstructive pulmonary disease	23	20
Chronic liver disease	9	7.8
Discharge	84	56
Death	66	44
ICU admission	122	81.3
MSI (mean ± SD)	1.41 ± 0.65	
q SOFA (mean ± SD)	1.41 ± 0.93	
**FOCUS OF INFECTION**		
Pulmonary	65	43.3
Genitourinary	58	38.7
Abdominal	16	10.7
Unknown	11	7.3

**Table 2:** Performance metrics of MSI and QSOFA for sepsis screening

**Statistic**	**MSI**		**q SOFA**	
	**Value**	**95% CI**	**Value**	**95% CI**
Sensitivity	91.76%	83.77% to 96.62%	84.52%	74.99% to 91.49%
Specificity	90.77%	80.98% to 96.54%	80.30%	68.68% to 89.07%
Positive Likelihood Ratio	9.94	4.63 to 21.36	4.29	2.61 to 7.04
Negative Likelihood Ratio	0.09	0.04 to 0.19	0.19	0.12 to 0.32
Positive Predictive Value	92.86%	85.81% to 96.54%	84.52%	76.89% to 89.97%
Negative Predictive Value	89.39%	80.50% to 94.51%	80.30%	70.92% to 87.20%
Accuracy	91.33%	85.64% to 95.30%	82.67%	75.64% to 88.35%
